# Multivisceral Transplantation for Diffuse Portomesenteric Thrombosis: Lessons Learned for Surgical Optimization

**DOI:** 10.3389/fsurg.2021.645302

**Published:** 2021-02-19

**Authors:** Emilio Canovai, Laurens J. Ceulemans, Nicholas Gilbo, Nicolas M. Duchateau, Gert De Hertogh, Martin Hiele, Ina Jochmans, Tim Vanuytsel, Geert Maleux, Marleen Verhaegen, Diethard Monbaliu, Jacques Pirenne

**Affiliations:** ^1^Department of Microbiology, Immunology and Transplantation, Katholieke Universiteit Leuven, Leuven, Belgium; ^2^Leuven Intestinal Failure and Transplantation Center, University Hospitals Leuven, Leuven, Belgium; ^3^Department of Abdominal Transplant Surgery and Transplant Coordination, University Hospitals Leuven, Leuven, Belgium; ^4^Laboratory of Respiratory Diseases and Thoracic Surgery (BREATHE), Chronic Diseases, Metabolism and Ageing, Katholieke Universiteit Leuven, Leuven, Belgium; ^5^Department of Thoracic Surgery, University Hospitals Leuven, Leuven, Belgium; ^6^Department of Pathology, University Hospitals Leuven, Leuven, Belgium; ^7^Department of Gastroenterology and Hepatology, University Hospitals Leuven, Leuven, Belgium; ^8^Department of Radiology, University Hospitals Leuven, Leuven, Belgium; ^9^Department of Anesthesiology, University Hospitals Leuven, Leuven, Belgium

**Keywords:** multivisceral transplantation, intestinal transplantation, portomesenteric and splenic venous thrombosis, embolisation (therapeutic), surgical technical improvement

## Abstract

**Background:** Multivisceral transplantation entails the en-bloc transplantation of stomach, duodenum, pancreas, liver and bowel following resection of the native organs. Diffuse portomesenteric thrombosis, defined as the complete occlusion of the portal system, can lead to life-threatening gastrointestinal bleeding, malnutrition and can be associated with liver and intestinal failure. Multivisceral transplantation is the only procedure that offers a definitive solution by completely replacing the portal system. However, this procedure is technically challenging in this setting. The aim of this study is to describe our experience, highlight the challenges and propose technical solutions.

**Materials and Methods:** We performed a retrospective analysis of our cohort undergoing multivisceral transplantation for diffuse portomesenteric thrombosis at our institution from 2000 to 2020. Donor and recipient demographics and surgical strategies were reviewed in detail and posttransplant complications and survival were analyzed.

**Results:** Five patients underwent MVTx. Median age was 47 years (23–62). All had diffuse portomesenteric thrombosis with life-threatening variceal bleeding. Major blood loss during exenteration was avoided by combining two techniques: embolization of the native organs followed by a novel, staged extraction. This prevented major perioperative blood loss [median intra-operative transfusion of 3 packed red blood cell units (0–5)]. Median CIT was 330 min (316–416). There was no perioperative death. One patient died due to invasive aspergillosis. Four others are alive and well with a median follow-up of 4.1 years (0.3–5.9).

**Conclusions:** Multivisceral transplantation should be considered in patients with diffuse portomesenteric thrombosis that cannot be treated by any other means. We propose a standardized surgical approach to limit the operative risk and improve the outcome.

## Introduction

**Figure 1:**
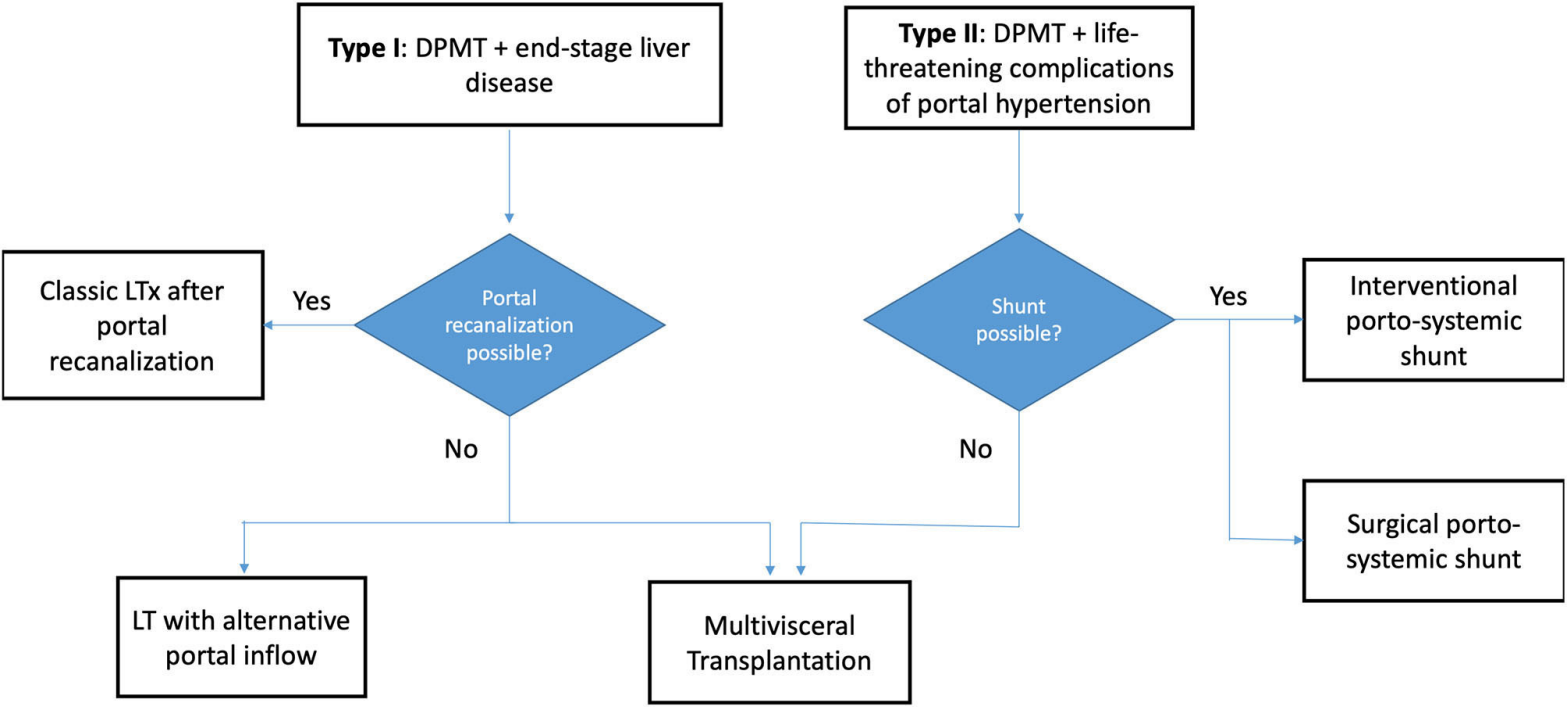
Flowchart demonstrating the various treatment options available for both DPMT types. DPMT, diffuse portomesenteric thrombosis; LTx, Liver Transplantation.

Multivisceral transplantation (MVTx) is defined as the exenteration of the native viscera followed by an en-bloc transplantation of stomach, liver, pancreas and small bowel. It is proposed as a radical therapeutic option for extensive abdominal pathology that is otherwise untreatable (1). In this procedure, a single cluster of organs is implanted on a combined arterial patch including celiac trunk (CT) and superior mesenteric artery (SMA) with venous outflow being provided through the inferior vena cava (IVC). Currently, the most frequent indication for MVTx is diffuse portomesenteric thrombosis (DPMT) (2). DPMT is defined as complete thrombosis of the portomesenteric vessels, resulting in severe portal hypertension and aberrant collateral circulation, with a major risk of gastro-intestinal bleeding (3). DPMT can present in two clinical forms which we propose to classify in Type I [associated with end-stage liver disease, seen in some liver transplant (LTx) candidates] and type II (no advanced liver disease justifying LTx, but life-threatening complications caused by portal hypertension) (Figure 1). The proportion of MVTx among all forms of intestinal transplantations (ITx) has increased up to 21% (4) but the survival remains inferior (5). The reasons for this are both medical and surgical. Medically, MVTx patients are often severely weakened in the pre-operative phase. After transplantation, they suffer from higher incidences of infection and graft-versus-host disease (GVHD) compared to other types of ITx (6). Surgically, MVTx represents the most invasive abdominal procedure that can be performed. In case of DPMT, MVTx is even more challenging due to the severe bleeding from the engorged collateral circulation that inevitably occurs during the resection phase (7). In type I DPMT, bleeding can be further exacerbated by liver disease-induced coagulopathy and severe adhesions. Over the last 10 years, survival rates have improved, mainly due to better patient selection, refinement in immunosuppressive protocols, use of extensive infectious prophylaxis (8), and accumulated surgical experience. However, MVTx is only performed in a few ITx centers worldwide and the technical aspects of the procedure have not been described in detail.

We aim to review our experience in MVTx, report technical challenges in detail, and propose surgical strategies to overcome them in order to improve the outcome.

## Materials and Methods

The study was a retrospective cohort analysis of a prospectively maintained database of ITx performed at the University Hospitals Leuven, Belgium between 01/01/2000 and 31/12/2020. Any patient receiving a MVTx transplantation was included. All patients remained in follow-up at our institution per protocol described elsewhere (9). Donor data included: age, gender, weight, cause of death, BMI, ABO, Cytomegalovirus status and days admitted. Recipient data included: age, gender, admission status, cause of DPMT, ABO compatibility, DPMT type, cold ischemia time (CIT), warm ischemia time, survival and outcomes including postoperative complications. Outcome data not already present in our prospective database, was extracted from patients' files maintained at our institution. These included medical and surgical complications. Complications were defined according to the Clavien-Dindo classification (10). All data is presented as median (range) unless stated otherwise.

### Patient Selection and Workup

**Figure 2:**
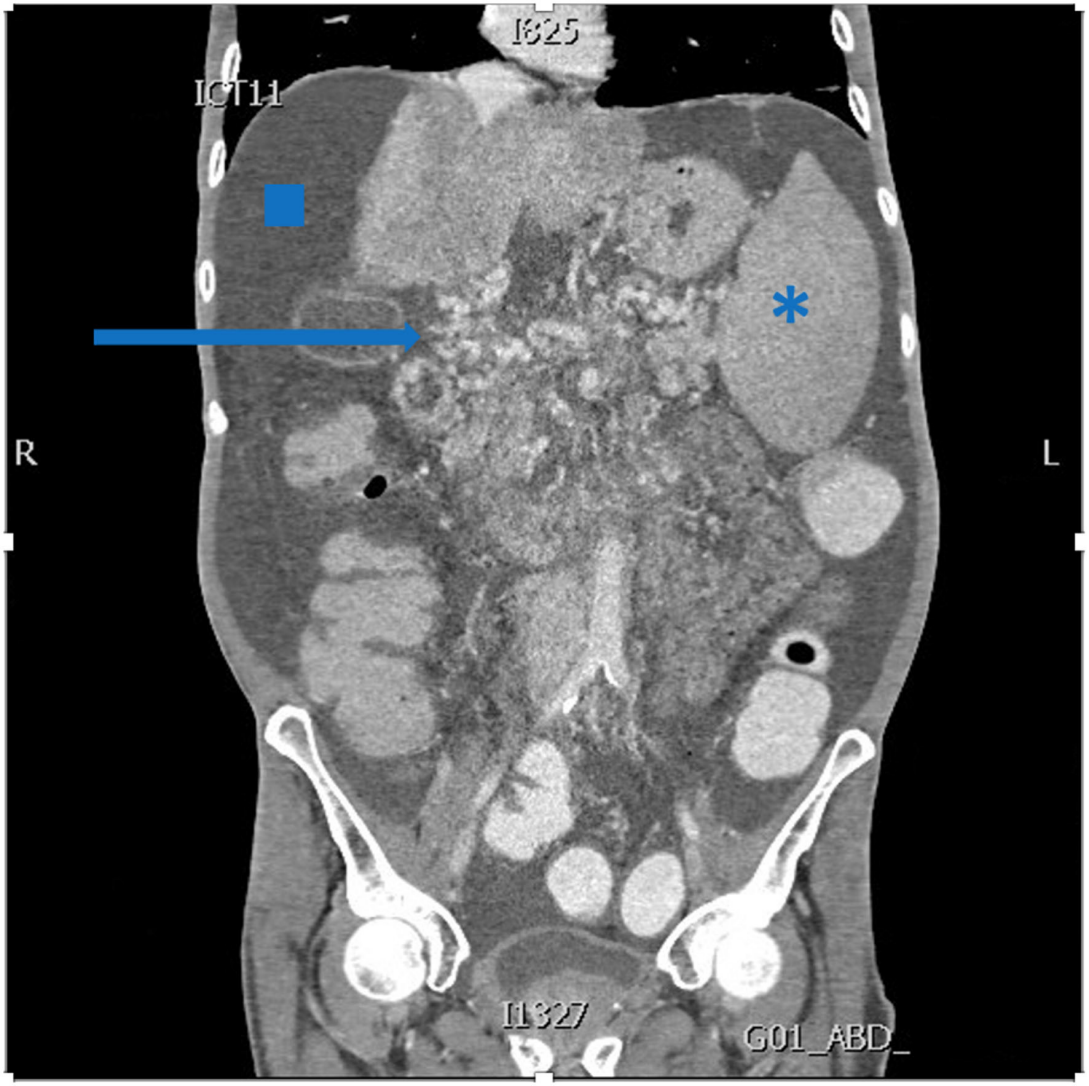
Abdominal computed tomography demonstrating extensive portal collaterals (arrow), ascites (square) and splenomegaly (asterisk).

Candidates for MVTx undergo an extensive workup, including a 3-phase computed tomography angiography (Figure 2), and their medical file is subsequently discussed at a multidisciplinary meeting. When considered not amenable to other medical, surgical and radiological decompressive treatments, a request for MVTx is submitted to Eurotransplant - to which Belgium participates. After approval, patients are listed and received priority immediately after the high-urgency LTx candidates, regardless of their model of end-stage liver disease (MELD) status.

Once a suitable donor is identified, both donor and recipient are pre-treated with the Leuven Immunomodulatory protocol described in detail elsewhere (9).

### Procedure

#### Organ Procurement

The donor is installed with both arms alongside the body. Since the abdominal domain in the recipient could be compromised by previous surgery or reperfusion edema of the graft, we start the procurement by dissection of the abdominal rectus fascia for an eventual non-vascularized fascia transplant. Our technique is based on the experience described by Gondolesi et al. (11). A median laparotomy is performed from the sternal notch to the pubic bone, followed by a dissection of the anterior fascia until the lateral edges so that the skin and subcutaneous tissue can be used to close the abdomen of the donor after the procurement procedure. Then a bilateral subcostal incision is performed and extended along the lateral edges of the rectus fascia and muscle up to the groin. This rectus wall is reflected caudally and covered in warm wet gauzes.

**Figure 3:**
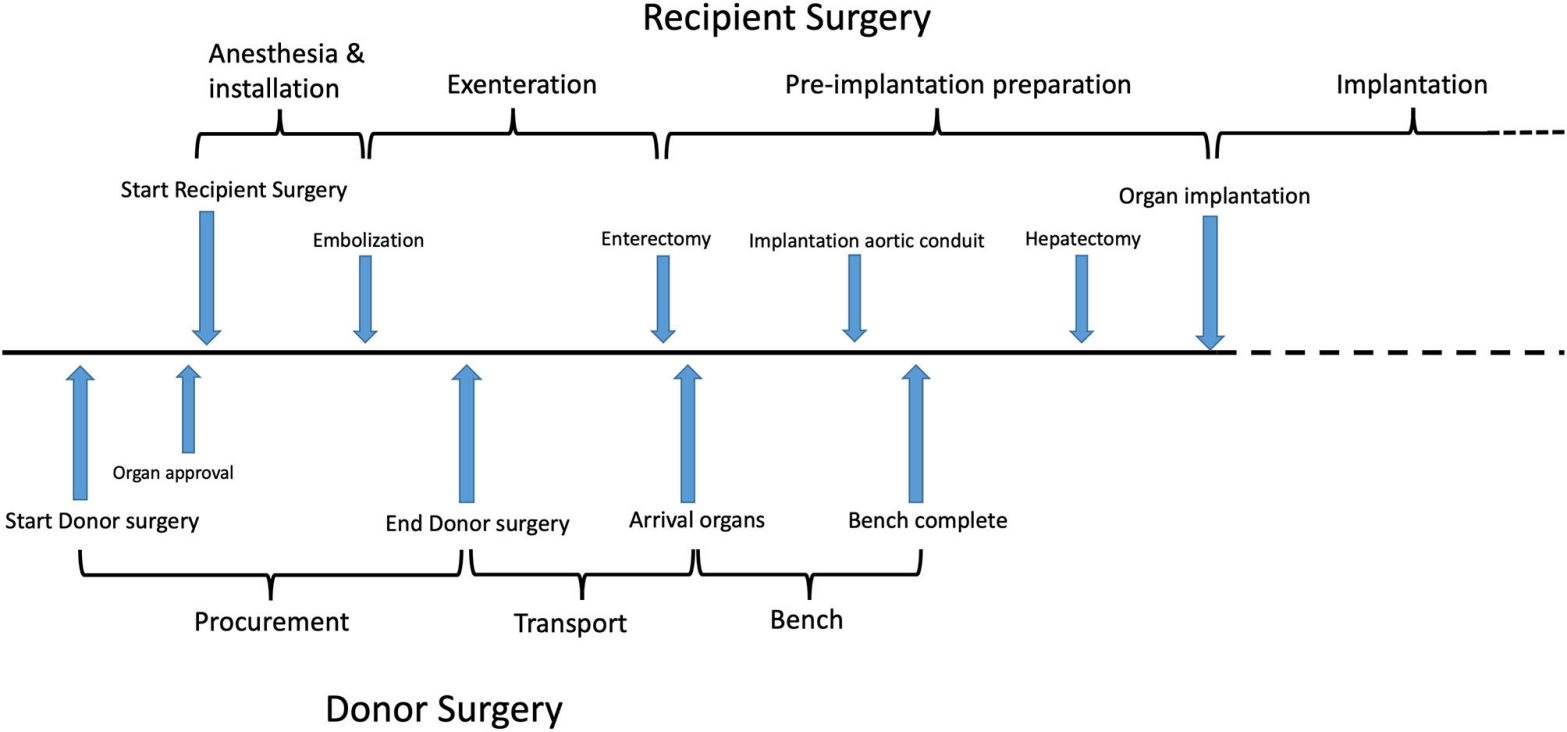
Timeline for both donor and recipient operations.

A median sternotomy is performed. After careful inspection and approval of the donor organs, the recipient is immediately prepared for surgery (see below). Minimizing CIT is essential requiring excellent coordination between the two teams (Figure 3). The right colon, duodenum and head of the pancreas are fully mobilized, exposing the aorta and the IVC. The SMA is identified and the surrounding tissue and lymph vessels carefully dissected and clipped. The aorta and IVC are then freed and encircled above the iliac bifurcation. The lesser omental sac is divided after verification of any accessory left hepatic artery. The abdominal esophagus and supra-celiac aorta are encircled. The spleen and tail of the pancreas are also mobilized. The colic arteries are identified and the ileocolic artery with its ileal branches is carefully preserved. After preparation of the thoracic organs, 300 International units/Kg of heparin are administered. The aortic cannula is inserted above the iliac bifurcation. Cold aortic perfusion is started with 6–7 liters of preservation solution [either University of Wisconsin or Institut George Lopez-1 (IGL-1)]. Venous drainage is established *via* the IVC and the organs are topically cooled. No separate portal or intestinal luminal flush is performed. After removal of the thoracic organs, the mesocolon is transected between the middle colic and ileocolic artery. Subsequently, the abdominal bloc (stomach, liver, pancreas, spleen, small bowel and ascending colon) is removed after transection of the thoracic and supra-renal aorta and IVC, and stapling of the distal esophagus and ascending colon (just above the caecum).

**Figure 4:**
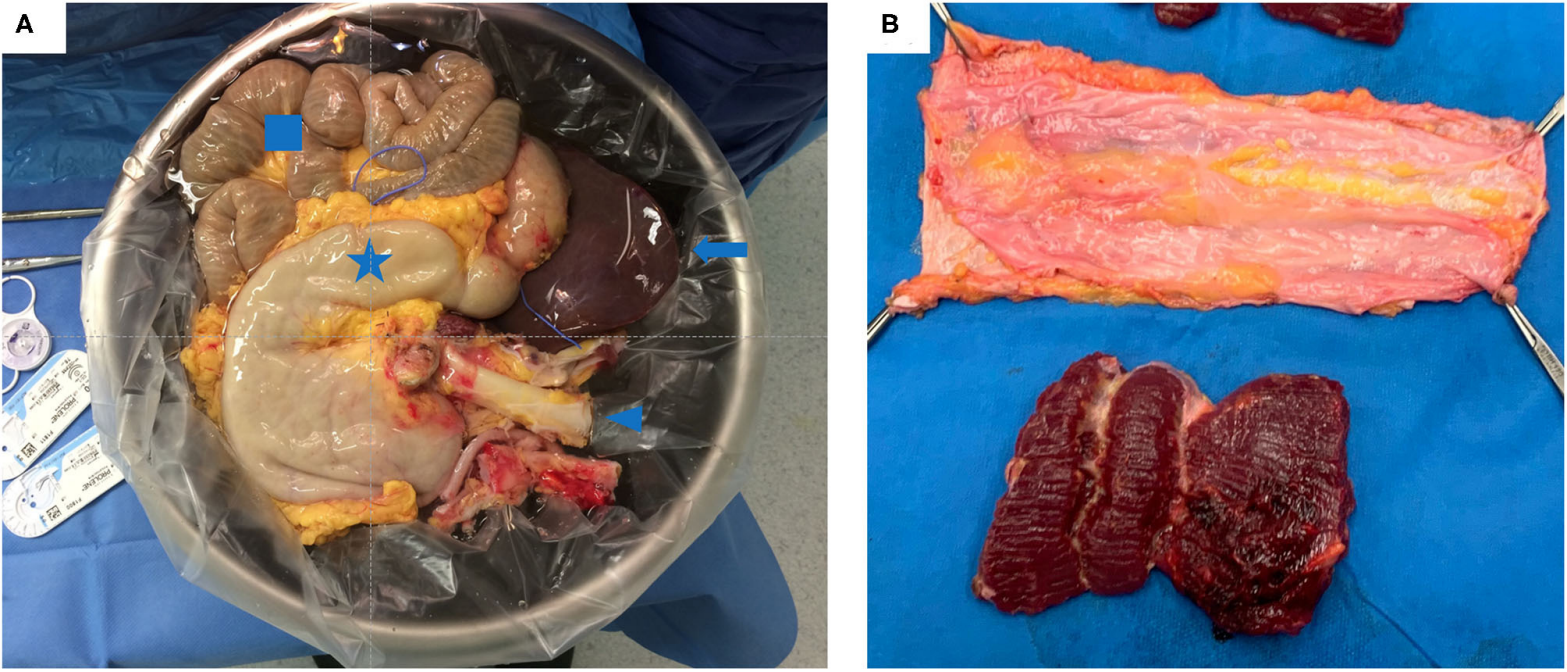
**(A)** Multivisceral graft on the back-table just prior to implantation. It contains: the stomach (star), liver (arrow), small bowel (square) and the aorta (arrow head) **(B)** A non-vascularized fascia graft after removing the rectus muscle.

After removal of the multivisceral bloc, the abdominal wall is cut and transferred to the bench table where the muscle is removed from the anterior and posterior rectus fascia (Figures 4A,B, respectively).

The multivisceral graft and non-vascularized fascia are packed separately and transported at 4°C in preservation solution.

#### Recipient Native Organ Embolization

**Figure 5:**
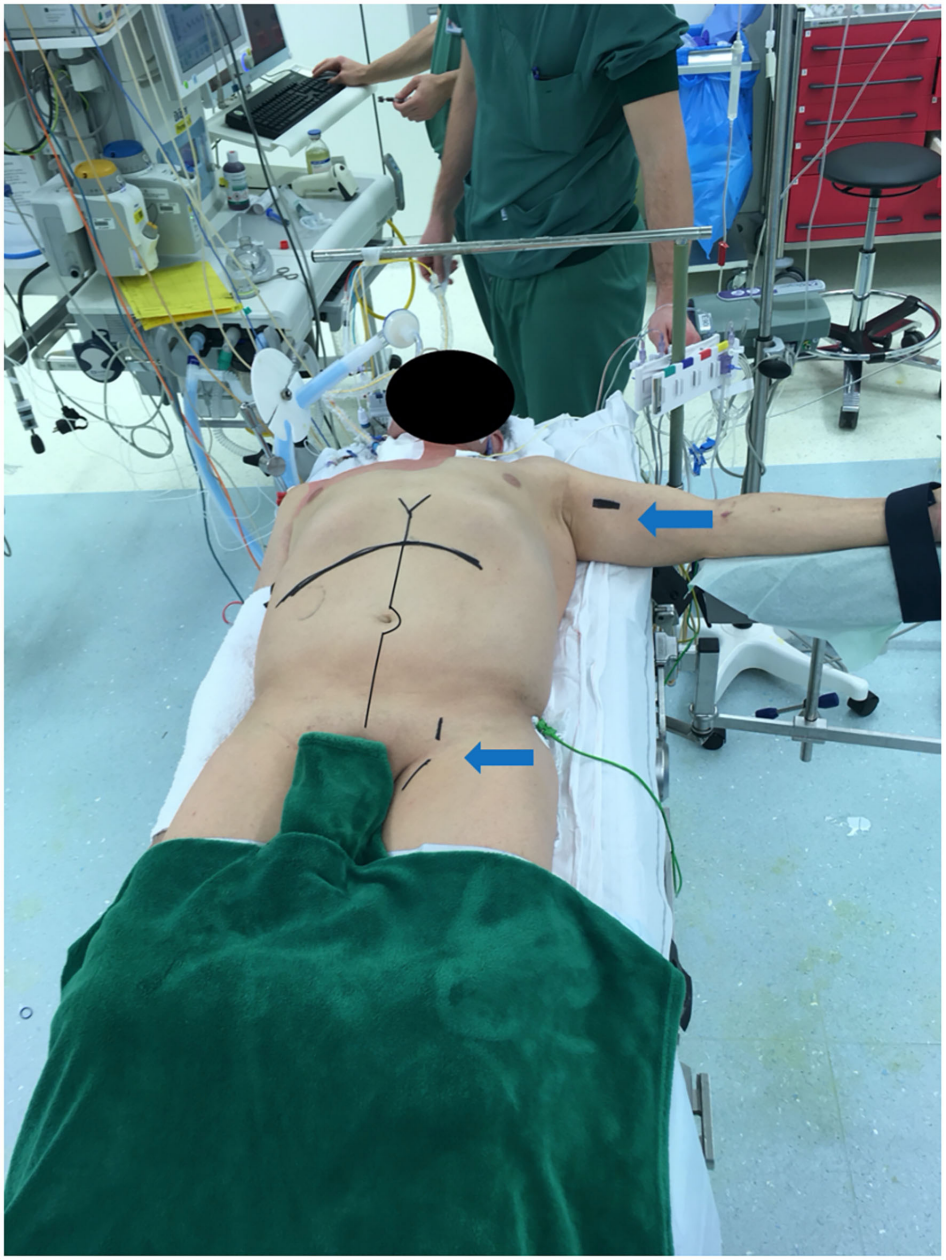
Recipient positioned for multivisceral transplantation. All patients receive a midline laparotomy and a subcostal incision to provide maximum exposure. Left arm and groin are also prepped for access for the venous-venous bypass (arrows).

**Figure 6:**
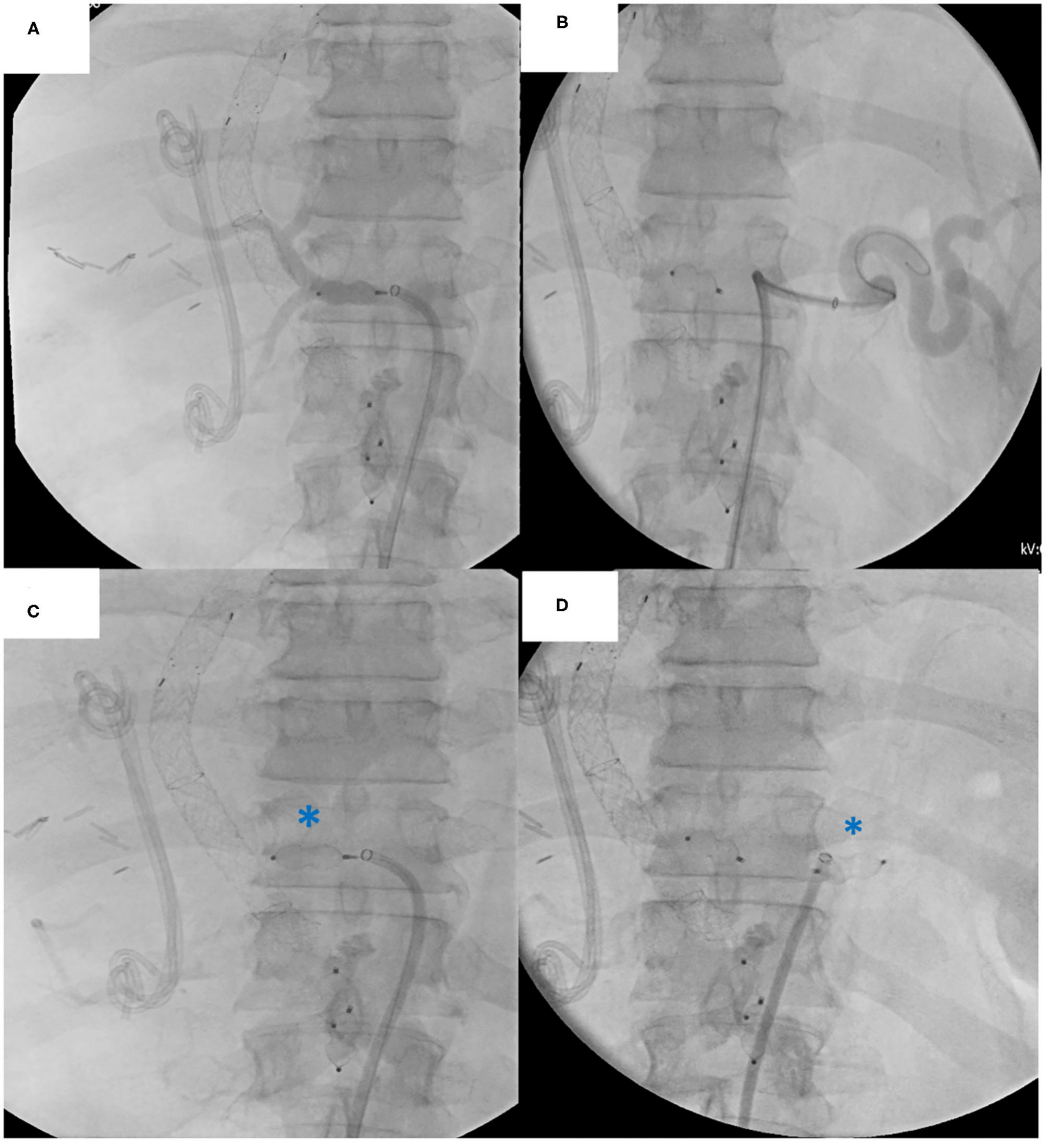
Native celiac trunk embolization procedure: **(A)** Angiography of the common hepatic artery **(B)** Angiography of the splenic artery **(C)** Plug embolization of the common hepatic artery **(D)** Plug embolization of the splenic artery. Note the biliary stent and TIPPS *in situ*.

**Figure 7:**
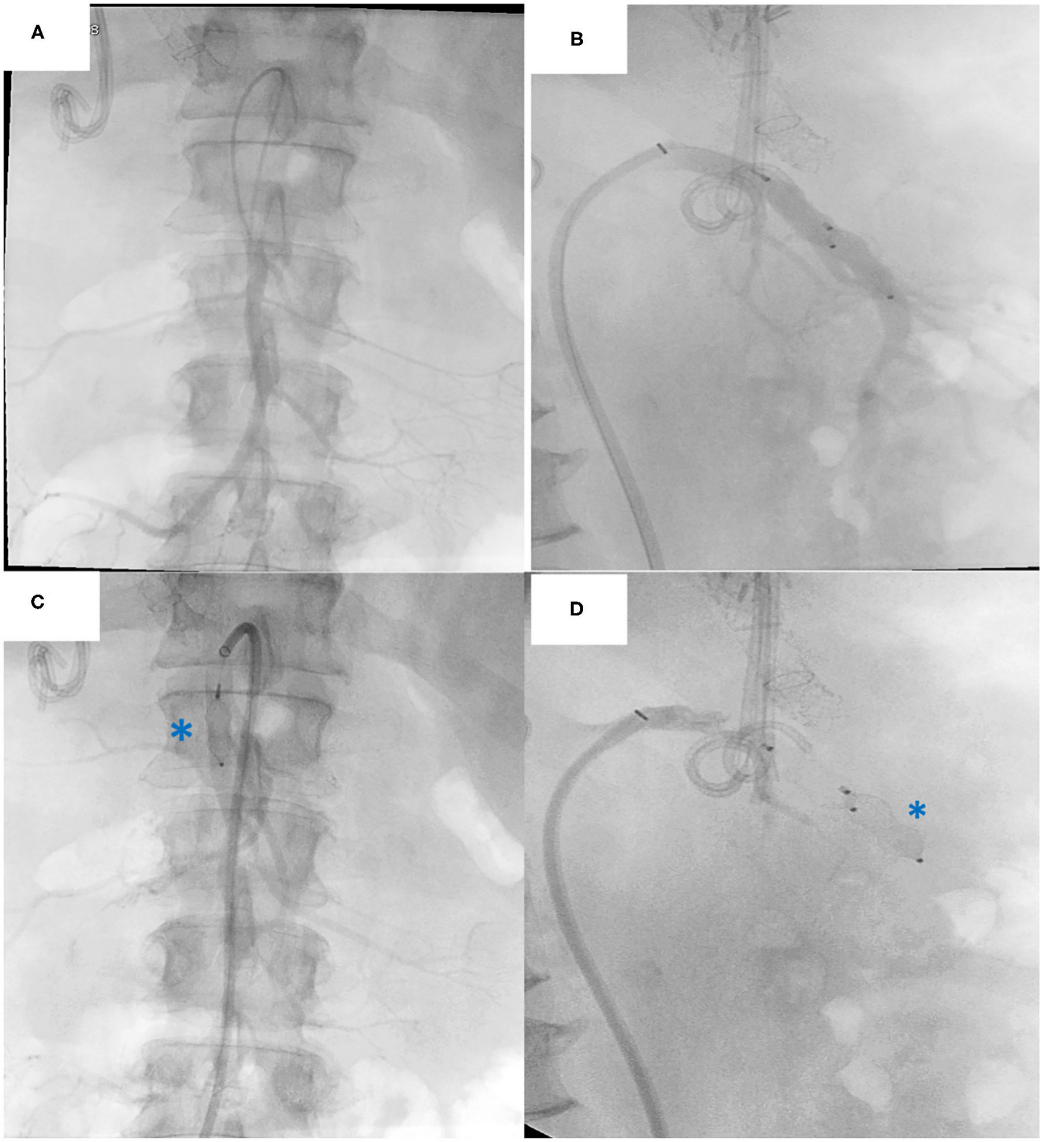
Native superior mesenteric artery (SMA) embolization procedure: **(A)** Frontal view Angiography of the SMA **(B)** Lateral view Angiography of the SMA. Plug embolization of the SMA **(C)** Anterior view of the plug (asterisk) **(D)** Lateral view of the plug (asterisk). Note the biliary stent and TIPPS *in situ*.

Immediately upon approval of the donor organs, the patient is anesthetized and installed with the right arm alongside the body and the left arm perpendicular to the body (Figure 5). The patient is scrubbed and draped from the lower thorax down to the groins. The most critical phase of the operation is the dissection and exenteration of the native organs which can provoke severe and life-threatening bleeding. To avoid this, we described a novel technique of pre-operative embolization of the CT and SMA (12). By interrupting the arterial inflow into the splanchnic viscera, the risk of bleeding is considerably reduced. The embolization is performed by the interventional radiologist using a mobile fluoroscopy C-arm (Arcadis^©^ Siemens, Erlangen, Germany). This occurs in the operating room after installing the recipient for surgery. After puncture of the right femoral artery, a vascular sheath is placed and a selective catheterization of the CT (Figures 6A,B), followed by the SMA is performed (Figures 7A,B). In the first three patients, polyvinyl alcohol microparticles [Contour^©^ (Boston Scientific)], or tris-acryl gelatin microspheres (Embosphere^©^ Merit Medical) and glue [mixture of Histoacryl^©^ (B. Braun) and Lipiodol^©^ (Guerbet)] were used as embolic agents. In the 4th and 5th patient, an oversized first generation Amplatzer^©^ vascular plug (St. Jude) was used (Figures 6C,D for the CT and Figures 7C,D for the SMA). For optimum effect, the timing relative to the exenteration and subsequent implantation must be perfect. We therefore start the embolization procedure around 2 h prior to the expected arrival of the donor organs. Given that the embolization lasts around 60 min, this provides sufficient time to start the laparotomy and perform the exenteration. This ensures the ischemic native organs stay in body for the shortest period possible. If transport time is short, the donor procedure will be delayed to allow sufficient time for the recipient procedure to progress further.

#### Bench Procedure

As the graft arrives, the thoracoabdominal aorta -still attached to the multivisceral bloc- is removed to be used as a conduit in the recipient (see below). A common circular arterial patch is created from the donor CT and the SMA. The donor IVC above and below the liver is freed to create length for the anastomosis. The three phrenic veins and the adrenal veins are tied. The spleen is removed and the peripancreatic tissue around the tail is tied off. The inferior mesenteric vein is cannulated to flush out the preservation solution during the implantation.

#### Recipient Native Organ Exenteration

Immediately after the embolization, the exenteration phase starts through a midline laparotomy and an additional bilateral cruciate incision to ensure excellent exposure. After extended mobilization of the right colon, duodenum and the head of the pancreas, the entire small bowel mesentery and the lateral side of the aorta as well as the origin of the CT and SMA are identified. They can be encircled and tied at this stage (if easily identified) or later (see below). The liver hilum is then tied off en-bloc and transected. After this, the left colon is mobilized and the sigmoid is stapled at the demarcation line between the devascularized area supplied by the embolized SMA and the area still perfused by the inferior mesenteric artery. At that stage, the hepatogastric ligament and the inner curvature of the stomach are transected until the esophago-gastric junction is seen. The latter is then resected paying attention to leave a small cuff of native stomach to facilitate the later upper GI reconstruction. The spleen and the tail of the pancreas are then mobilized medially until the left side of the aorta and the CT and SMA become exposed. If not yet divided, the CT and SMA can now be tied either individually or sutured en-bloc. The gastrointestinal bloc is removed while the native liver remains *in situ* at this stage.

#### Multivisceral Bloc Implantation

**Figure 8:**
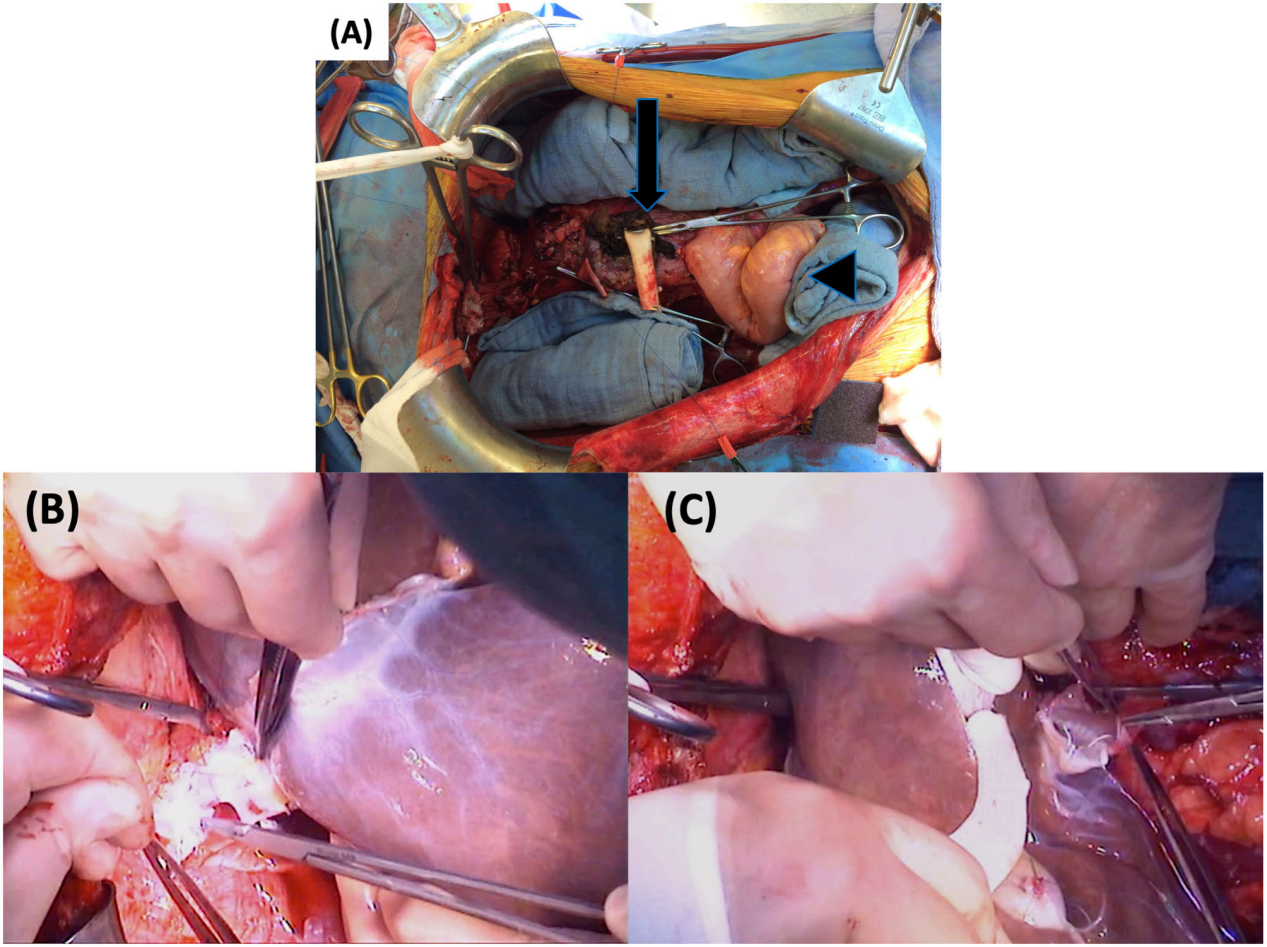
First part of graft implantation: **(A)** Empty abdomen prior to implantation with the aortic conduit (arrow) and the native sigmoid (arrowhead) **(B)** Supra-hepatic IVC anastomosis **(C)** Infra-hepatic IVC anastomosis. IVC, inferior vena cava.

**Figure 9:**
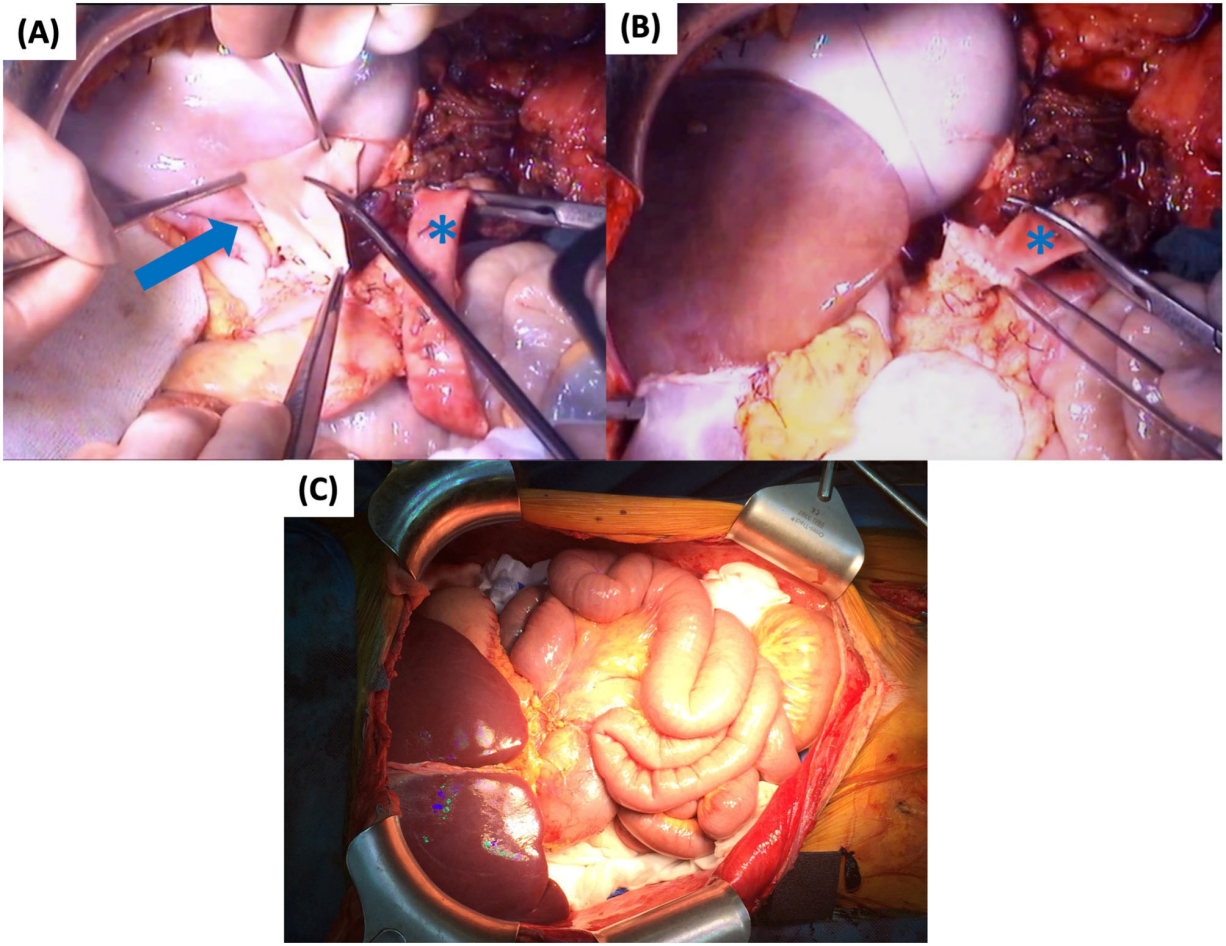
Second part of graft implantation: **(A)** Preparation of common CT and SMA patch (arrow) **(B)** Arterial anastomosis to the conduit (asterisk) **(C)** Graft reperfusion. CT, celiac trunk; SMA, superior mesenteric artery.

First, the donor aortic tube is implanted end-to-side on the infra-renal aorta using 5/0 Prolene^©^ (Ethicon). Next, a veno-venous bypass (from femoral vein to left axillary vein) is installed and the native liver is removed after clamping the IVC above and below the liver (Figure 8A). The multivisceral graft is then implanted using the caval replacement technique by an end-to-end IVC anastomosis first above (Figure 8B) and then below the liver (Figure 8C). These anastomoses are performed with running 5/0 Prolene^©^ suture. Next, an end-to-end arterial anastomosis is performed between the donor aortic patch (including CT and SMA) and the aortic conduit using 6-0 Prolene^©^ (Ethicon) (Figures 9A,B). During implantation, the graft is continuously topically cooled and the preservation solution is flushed out using a colloid solution (SOPP-SSPP4%) administered through the inferior mesenteric vein. After the IVC clamps are released and any bleeding is controlled, the aortic clamp is released and the graft is reperfused (Figure 9C). After hemostasis and hemodynamic stabilization, the veno-venous bypass is stopped. A short reperfusion break of 15 min is taken. Next, a cholecystectomy is performed. Proximal intestinal continuity is then restored, either by a gastro-gastric or esophago-gastric anastomosis. Since the stomach is denervated, a pylorotomy is performed to prevent gastric outlet syndrome. To avoid gastroesophageal reflux and to protect the proximal anastomosis, a Nissen fundoplication is performed. Distally, a colo-colic (*n* = 1) or ileocolonic (*n* = 4) anastomosis is performed. All intestinal anastomoses are performed manually in two layers: Vicryl^©^ 3/0 (Ethicon) continuous submucosal suture line followed by interrupted serosal sutures with Prolene^©^ 4-0. Depending upon the nutritional state, a feeding gastrostomy and/or jejunostomy is placed. Finally, a double loop distal ileostomy is externalized in the right lower quadrant to protect the distal anastomosis and allow easy access for endoscopy. After extensive abdominal lavage with 10 liters of warm physiological solution and placement of 4 drains (two on the right side and two on the left side; one of them close to the aortic tube), the abdomen is closed (primarily in all patients) and the ileostomy is matured.

### Post-operative Management

All patients receive broad spectrum antibiotic/antifungal prophylaxis including piperacillin/tazobactam and amphotericin B for 1 week and fluconazole for 3 months. Ganciclovir is given irrespective of cytomegalovirus status and co-trimoxazole is given for Pneumocystis Jirovecii profylaxis. The immunosuppressive therapy and posttransplant protocol have been described previously in detail (9).

## Results

Out of 22 ITx patients performed at our center between 2000 and 2020, 11 (50%) were isolated ITx, 6 (27%) were combined liver bowel transplants, and 5 patients underwent a MVTx (23%). Age at time of MVTx was 47 years (23–62) with a BMI of 24 kg/m^2^ (15–33). They were all male. All had symptomatic DPMT -Yerdel grade 4 (3)- with recurrent life-threatening gastrointestinal bleeding and portal congestion. Underlying causes are listed in Table 1. Two patients were hospitalized at the time of transplantation (Patient 1: upper gastrointestinal bleeding and Patient 3 for liver decompensation). Patient 2 additionally had intestinal failure and recurrent ascites. The lab-MELD score was 13 (10–32). Patient 3 had severe liver disease (Type I DPMT) while the other patients did not (Type II DPMT). Patient 3 also had severe renal failure. All patients received a graft from an ABO identical or compatible brain-dead donor. Additional donor data is listed in Table 2. All donors underwent a standardized pre-treatment protocol described elsewhere (9). University of Wisconsin preservation solution was used in the first 3 donors (6–7 liters) while institute George Lopez-1 preservation solution was used in the last two donors (6 liters) (13). The CIT was 331 min (316–416).

**Table 1:** Recipient characteristics.

**Recipient**	**Age (years)**	**Gender**	**Location at time of MVTx**	**Cause of DPMT**	**Indication for MVTx**	**ABO compatibility**	**DPMT type**	**CIT (minutes)**	**WIT (minutes)**	**Survival (days)**	**Outcome**
1	43	Male	Hospitalized	Antiphospholipid syndrome	Recurrent GI bleeding	Identical	2	395	38	254	Died - invasive aspergillosis
2	23	Male	Home	Pancreatic NET with liver metastasis	Recurrent GI bleeding -Intestinal Failure -Ascites	Identical	2	316	24	2167	Alive – HPN independent with treated NET recurrence
3	47	Male	Hospitalized	Alcohol-induced cirrhosis	-Liver decompensation -Renal failure	Compatible	1	330	30	2104	Alive – HPN independent
4	47	Male	Home	Unidentified protrombotic syndrome	Recurrent GI bleeding	Identical	2	331	44	879	Alive - HPN independent
5	62	Male	Home	Portal hypertension of unknown origin	Recurrent GI bleeding, recurrent cholangitis	Identical	2	416	38	213	Alive – HPN independent

*CIT, Cold ischemia time; DPMT, Diffuse portomesenteric thrombosis; GI, Gastrointestinal; HPN, Home parenteral nutrition; MVTx, Multivisceral transplantation; NET, Neuroendocrine tumor; WIT, Warm ischemia time*.

**Table 2:** Donor characteristics.

**Donor**	**Age (years)**	**Gender**	**Cause of death**	**BMI (kg/m^**2**^)**	**ABO type**	**CMV status**	**ICU admission time (days)**
1	16	Male	Head trauma	20	O+	–	4
2	21	Male	Intracranial bleeding - ruptured aneurysm	20	O+	–	1
3	31	Male	Head trauma	26	A–	+	4
4	28	Male	Head trauma	20	A–	+	3
5	15	Female	Suicide (hanging)	20	A+	–	3

*BMI, Body mass index; CMV, Cytomegalovirus; ICU, Intensive care unit; NET, Neuroendocrine tumor*.

All embolization procedures were uneventful. Embolization in the first 3 patients (using embolic agents) took 80 min (70–90). Plug embolization of the CT and SMA in the last two patients was performed in 35 and 50 min, respectively.

The median graft implantation time (warm ischemia) was 38 min (24–44). In patient 1, a cuff of native stomach was left in place and a proximal gastro-gastric anastomosis was performed. A segment of ascending colon was transplanted and a distal colo-colic anastomosis was performed. In the other patients, an esophago-gastric anastomosis was performed proximally and an ileocolonic anastomosis distally without transplanting colon. There were no intraoperative deaths. Intra-operative transfusion requirement was 3 units of blood (0–5), despite low pretransplant hemoglobin levels of 7.4 g/dL (5.6–10.7). The first 3 patients received a gastrostomy and jejunostomy while the last two patients only received a nasogastric tube. All patients received a double loop ileostomy in the right lower quadrant. In all recipients, primary closure was possible and thus no rectus fascia was used. At the end of the MVTx, patient 3 received an additional kidney transplantation placed retroperitoneally in the left lower quadrant through a separate hockey stick incision using standard technique. Total recipient procedure time was 588 min (510–780).

### Rejections

Two of our patients (Patients 2 and 4) had an asymptomatic grade I rejection diagnosed at protocol biopsy on day 20 and 26, respectively, and responded well to high dose corticosteroids. Patient 5 had an asymptomatic grade II rejection on protocol biopsy on day 25, which also responded well to corticosteroids. Patient 1 developed a symptomatic (diarrhea, unwell) grade III rejection that was refractory to corticosteroids, sirolimus and Muromonab-CD3. He required a partial graft enterectomy on day 67. Eventually the patient developed multifocal invasive aspergillosis which led to intracerebral bleeding due to cerebral mycotic aneurysm rupture. The patient died on day 254 post-transplant.

### Surgical Complications and General Outcomes

The 5 patients had 8 surgical complications ranging from Clavien-Dindo class II to IV (Table 3) (10).

**Table 3:** Post-operative complications (first year) scored according to Clavien-Dindo classification.

**Patient number**	**Medical complications**	**Surgical complications**	**Surgical Treatment**
1	Refractory Grade III rejection (CD V)	Ischemic colitis (CD IIIb)	Partial colectomy of the native colon
2	Grade I rejection - asymptomatic (CD II)	Volvulus of the distal ileum around ileostomy (CD IIIb)	Partial enterectomy and new ileostomy
3	Lung infection with *Nocardia asteroides* (CD II)	Leakage at ileocolic anastomosis and bleeding from arterial conduit (CD IV)	Oversuturing bleeding, partial graft resection and re-anastomosis
	Renal transplant AKI (CD II)	Mycotic aneurysm arterial conduit (CD IIIb)	Replacement with aortic/iliac homograft
4	Grade I rejection – asymptomatic (CD II)	Endovascular plug migration into the aorta (CD II)	None
	Bilateral Lung embolism (CD II)		
5	Liver graft dysfunction (CD IV)	Stomal retraction (CD IIIb)	Partial distal enterectomy and new ileostomy
	AKI (Hemodialysis) (CD II)	Asymptomatic SMA stenosis (CD IIIa)	PTAS of SMA
	Grade II rejection - asymptomatic (CD II)	Bleeding from jejunal ulcer (CD IIIb)	Partial jejunal resection

*AKI, Acute kidney injury; CD, Clavien-Dindo classification of complications; PTAS, percutaneous transluminal angioplasty and stenting; SMA, superior mesenteric artery*.

**Figure 10:**
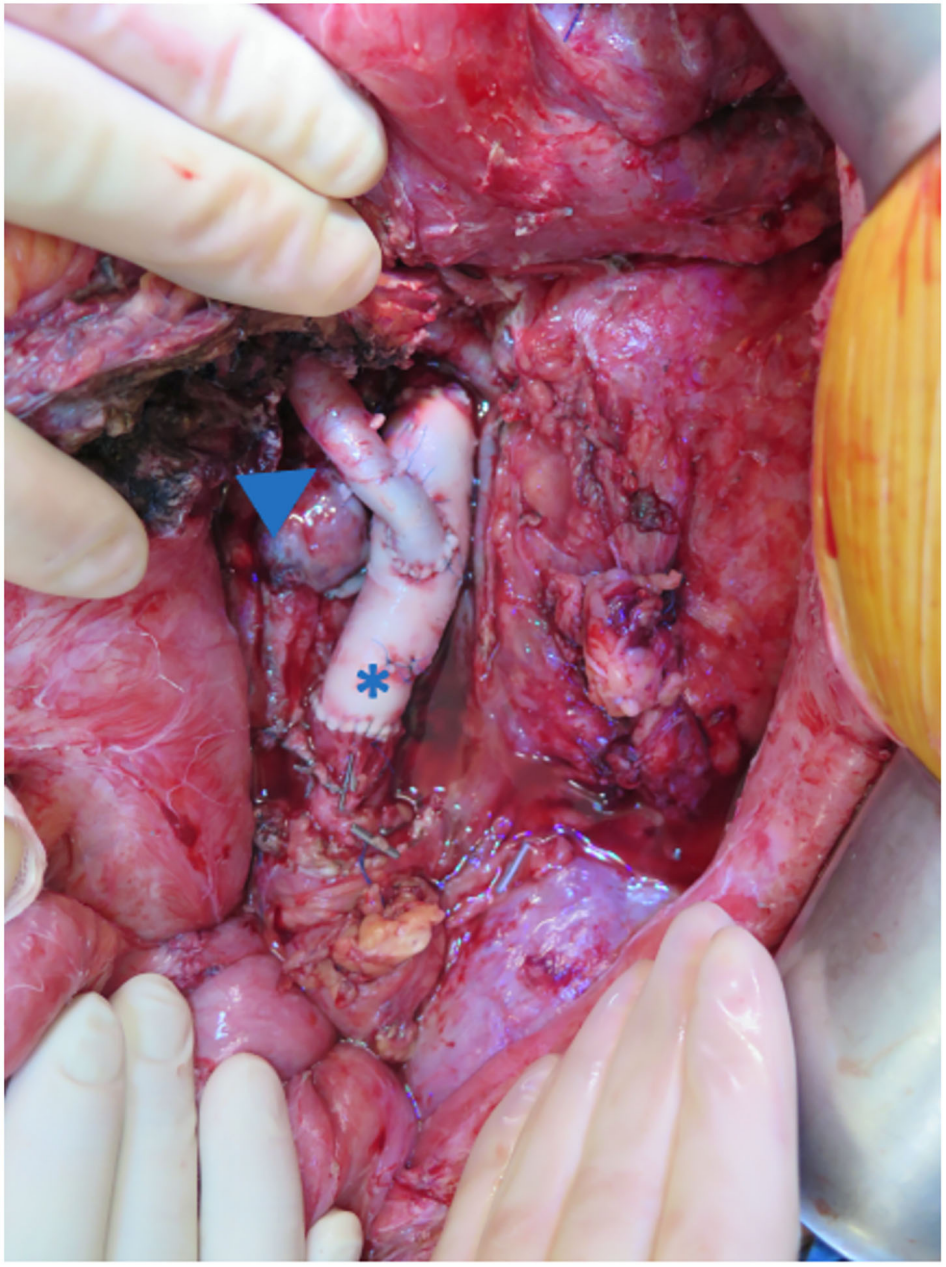
The infra-renal aorta was replaced with an aortic homograft (asterisk). The aortic conduit was reconstructed using an iliac homograft (arrow).

Of interest, patient 3 developed a mycotic pseudo-aneurysm of the native aorta and aortic conduit, probably secondary to the previous intestinal contamination. The infected infra-renal aorta and the conduit were resected and reconstructed using allografts (14) (Figure 10).

**Figure 11:**
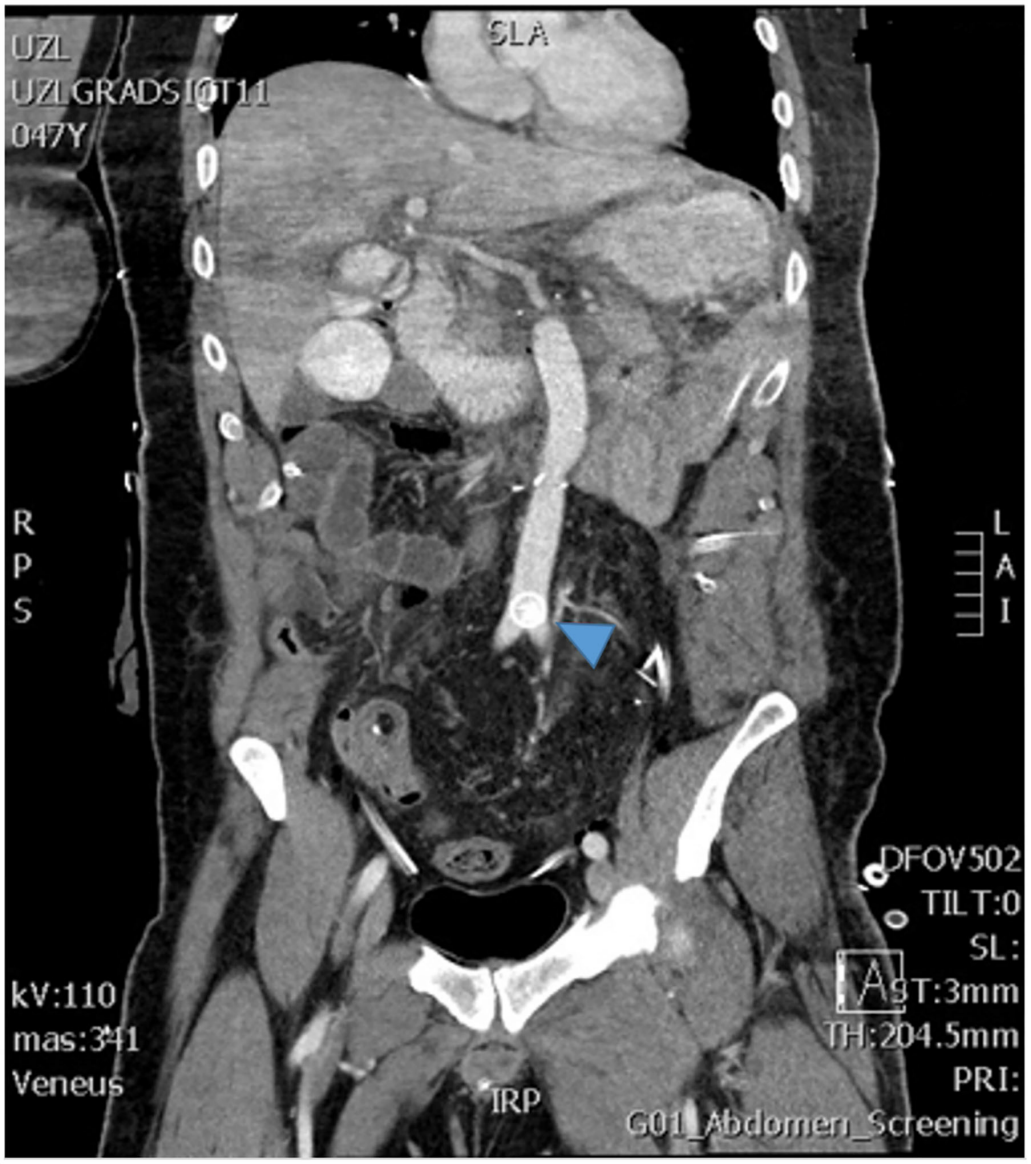
The postoperative imaging shows the vascular plug that dislocated from the superior mesenteric artery and migrated to the aortic bifurcation (arrow).

In patient 4, an asymptomatic dislodgement of the SMA vascular plug was detected during routine imaging (Figure 11). As the patient was completely asymptomatic, this was treated conservatively with aspirin. There was no clot formation around the plug on imaging, nor have there been signs of distal embolization.

Furthermore, patient 2 developed a metastasis 2 years after MVTx of the neuro-endocrine tumor in the thorax, liver and bone, which responded well to chemotherapy (oxaliplatin, fluorouracil), polypeptide receptor radionuclide therapy, and m-TOR inhibitor. He is well 6 years posttransplant.

All surviving patients (4/5) are alive with functioning grafts at home [follow-up 4.1 year (0.6–5.9 years)].

## Discussion

MVTx involves a resection of all splanchnic organs followed by en-bloc transplantation. The technique was first developed in animal models by Starzl et al. in the 1960s (15, 16) and was then applied in man for the first time in Pittsburgh by Starzl et al. (1) and in Chicago by Williams et al. (17) in 1989.

Since then, untreatable DPMT has been the leading indication for MVTx (2). Although experience has been expanding in the last 2 decades (5, 6), the first large series for MVTx for DPMT was only recently described in 25 patients (7). The reported 72% 5-year survival was encouraging despite a high complication rate. In the present case series, we describe in detail the techniques used for MVTx for DPMT, and propose potential solutions for the encountered surgical problems.

The most challenging part of MVTx is the removal of the native organs, particularly in DPMT due to the massively congested collateral circulation (8). In the largest series described so far, Vianna et al. reported a median peri-operative blood transfusion of 29 units (5–146) (7). To limit bleeding, the arterial inflow through the CT and the SMA should be suppressed as soon as possible. The difficulty is that this exposure requires extensive mobilization of the viscera. This can in itself cause severe blood loss and hemodynamic instability. To accelerate access to the CT and SMA, some authors recommend transecting either the transverse colon and pancreas (2) or the esophagus (18) followed by mass clamping of vascular inflow, but this too can cause major bleeding.

The major advantage of our pre-operative embolization technique is that complete suppression of arterial flow is obtained before surgery starts. Because the portal circulation is interrupted, the abdomen becomes a virtually bloodless field (12). This was demonstrated previously in cirrhotic patients, where temporary occlusion of splanchnic arterial inflow greatly reduced portal pressure gradients (19).

One limitation of the embolization technique in type 1 DPMT (Figure 1) is that it does not allow an attempt at an isolated liver transplantation (LTx). With the back-up of a MVTx graft Vianna et al. were able to perform an isolated LTx in 6 out of 30 cases (19%) using either thrombo-endovenectomy or venous jump graft (7). The remaining intestinal graft was then transplanted into another recipient. In our patients however, pretransplant imaging showed chronic, complete portal vein thrombosis (i.e., Yerdel grade 4, including the splenic vein) which would have made a standard LTx impossible (3, 20). The only remaining option would have been a cavo-portal transposition, a procedure that does not resolve portal hypertension and leads to persisting gastrointestinal bleeding and ascites (20, 21).

In case of type II DPMT (Figure 1), an isolated LTx would not be appropriate since liver function is usually next to normal. In these cases, MVTx is the only therapy to replace the “failing portal system” if it cannot be decompressed by interventional or surgical shunts (22, 23).

In our first 3 patients, we used a mixture of embolic agents to embolize the CT and SMA. It took up to 90 min to completely occlude all vascular side branches. In order to shorten the embolization time, the Cambridge team modified our technique and used vascular plugs instead of embolic agents (24). A vascular plug may also allow a more selective embolization, for example sparing the left gastric artery (or even the hepatic artery to preserve the stomach and the liver in modified MVTx). One risk, however, is the migration of a plug into the aorta as we faced in patient 4 (Figure 11). Plug migration has previously been described after other embolization procedures (25, 26) and this risk is particularly high when combined with surgical manipulation (27). In our patient we opted for a conservative therapy because it was a wide meshed plug (first generation Amplatzer^©^) in a high flow location without hemodynamic or embolic complications. In case endovascular plug extraction is considered, it should be done early after implantation (within 3 days). At a later stage, surgical extraction would be the only option (25).

The Miami group recently performed preoperative embolization in 3 MVTx in patients with a hostile abdomen (28). The first patient died intraoperatively after a difficult and lengthy exenteration and mass transfusion related coagulopathy (97 units transfused). Distal migration of the plug into the gastroduodenal artery resulting in re-arterialization of the liver may also have played a role. To avoid plug migration for their next two cases, they switched to distal embolization using embolic agents. Furthermore, they also spare the hepatic artery to shorten the anhepatic phase, and the left gastric artery to prevent gastro-gastric anastomotic leaks. However, sparing tributaries of the CT (or the SMA) maintains a persistent arterial and portal flow and may reduce the hemostatic effect of the embolization (29).

We feel that the risk of migration does not outweigh the benefits of a shorter embolization time. In our last case, the risk of intra-aortic migration was minimized by placing the plug slightly more distally and by clamping the CT and SMA proximal to the plugs. In addition, we now verify the position of the plugs fluoroscopically at the end of the transplantation. In case of plug migration, it can then be removed endovascularly (30) or through an arteriotomy (25).

Most centers remove the native viscera including the liver, in one bloc. In contrast, our exenteration method proceeds in two-steps. We first transect the liver hilum en-bloc (a maneuver without risk of portal congestion due to the preoperative embolization) and then remove the gastrointestinal tract (stomach, duodenum, pancreas, spleen, and intestines) while leaving the liver *in situ*. We then implant the donor aortic tube onto the infra-renal aorta, while the devascularized liver is still in place. When the graft is ready for implantation, we start veno-venous bypass and the liver is removed. The MVTx graft can then be implanted. This two-step exenteration technique and veno-venous bypass preserves normal systemic venous flow and hemodynamic stability.

For implantation, we use the caval replacement technique instead of the more commonly used “piggy back” technique. Both techniques have been used in isolated LTx and there is no evidence for the superiority of either (31). However, in the context of MVTx we believe that the classical caval replacement technique has the potential advantage to “anchor” the graft and prevent retrohepatic IVC rotation which is known to occur after MVTx (32).

For arterial inflow, we anastomose the donor patch end-to-end to the already placed infra-renal aortic conduit (Figures 8A,B). Arterial implantation onto the supra-celiac aorta either directly or through a conduit has also been described (18). The advantage of the infra-renal implantation is that this area is easier to reach and renal ischemia is avoided. In LTx, these conduits have a high thrombosis risk because blood must flow against gravity (33). However, in MVTx the outflow resistance is much lower, making these conduits less prone to thrombosis. Indeed, arterial thrombosis occurs in only 2.8% of all ITx compared to 9% in LTx alone (34, 35).

The arterial conduit is prone to infections, given that some intestinal contamination is inevitable. In our series, patient 3 developed a mycotic aneurysm after 5 months. This was corrected before any rupture could occur using aortic and iliac arterial homografts (14) (see Figure 10). The reported incidence of mycotic aneurysms was 2.5% in a large series of composite visceral allografts (*n* = 7/285) (34). Unlike in our case, most patients presented with an acute rupture and hemodynamic instability. In this setting, temporary endovascular occlusion can stop the bleeding and allow sufficient time for surgical repair (34). However, despite prompt treatment, this complication is often fatal. In an attempt to reduce perioperative contamination, we now perform extensive abdominal lavage (10 liters of warm physiological solution) with antibiotics (Meropenem) and antifungal medication (Amphotericin B) at the end of the procedure. Also, all patients receive 1 week of antibiotic prophylaxis.

Needless to say, construction of well vascularized intestinal anastomoses is crucial. The first patient developed a leak at the gastro-gastric junction, possibly secondary to the ischemia of the remaining cuff of native stomach, inherent to the complete embolization of the CT. Since then, we remove the native stomach completely and we perform an esophago-gastrostomy. An alternative to keep the upper part of the native stomach well vascularized would be to selectively embolize the CT and spare the left gastric artery (12, 28).

To prevent gastric outlet obstruction and gastroesophageal reflux in a denervated stomach, a pyloromyotomy and a Nissen fundoplication are performed. Additional advantage of the later is that it also protects the proximal esophago-gastric anastomosis.

Obliteration of the CT and SMA (by tying or embolization) leaves the inferior mesenteric artery open and the sigmoid and rectum remain vascularized. It remains important to transect the sigmoid well distal from the demarcation line and allow a safe margin to avoid anastomotic ischemia and leakage as seen in patient 1.

A few centers advise the transplantation of the colon, either partially or completely, to augment the gastrointestinal resorptive function (36). This trend is seen globally in the ITx registry and seems to result in less episodes of dehydration and faster weaning from parenteral nutrition (4). However, our patients had a sufficient length of native sigmoid and rectum, obviating the need for additional colon transplantation and its associated morbidity (37, 38).

At the end of the procedure, a distal ileostomy is routinely constructed in order to provide easy access for endoscopic biopsies. We prefer a loop ileostomy that can be closed easily after a few months. In an effort to reduce ostomy-related morbidity, the Miami group either creates an hybrid ostomy using an excluded ileal segment or avoided an ostomy altogether (36). However, this limits access to the graft for surveillance and may put the distal ileocolonic anastomosis at risk for leakage.

Essential in the success of MVTx is the maintenance of hemodynamic stability during the entire procedure. Firstly, limiting the exenteration/ischemic phase is crucial to reduce the risk of acidosis and metabolic instability. The use of intra-operative renal replacement therapy has been advocated by Cambridge and Miami to eliminate circulating waste products from ischemic organs during exenteration, and better maintain metabolic homeostasis until the new graft is functioning properly (24, 28). Equally important to the success of the procedure, is a short CIT (ideally <5 h). The recipient should be anesthetized immediately after approval of the donor organs. The patient should not remain anhepatic for too long (ideally <2 h). For this, a perfect coordination between donor and recipient team is essential.

The inclusion of the spleen in the MVTx graft and the fate of the native spleen is another contentious issue. Proponents of transplanting the spleen point to its potential to reduce rejection and infections, especially in children (39, 40). However, transplanting the spleen increases the risk of GVHD (41). The Madrid team preserves the native spleen without transplanting the donor spleen in children (42). This has the theoretical advantage of preventing infections while reducing the risk of GVHD. However, not all patients have a functional spleen and its preservation can substantially increase exenteration time. In the setting of DPMT, spleen preservation is virtually impossible. At our center, both native and donor spleen are removed. To prevent infections, we vaccinate our patients for *S. pneumoniae, N. meningitidis, H. influenzae* type b and influenza virus prior to MVTx (43). No infections or thrombosis associated with asplenia were seen in our patients. We also did not see GVHD or lymphoproliferative disorders.

The intestine is extremely immunogenic and prone to rejection (44). However, the risk of severe rejection after MVTx is lower compared to isolated ITx due to the immunoprotective effect of the liver (5, 8). In our series, only one patient had a severe grade III rejection. Early asymptomatic rejection was detected on protocol biopsy in 3 patients but could easily be controlled with corticosteroids.

Several limitations with this study have to be acknowledged. As this is a rare entity, the numbers are low which makes generalization difficult. Also, this study limits itself to one indication of MVTx, namely DPMT, which is also the most challenging one. Certain surgical aspects, such as the embolization, are specifically useful in this category and may not be extrapolated to all MVTx procedures. Only patients that actually underwent MVTx (after multidisciplinary approval) were included in the study which leads to an inevitable selection bias.

In conclusion, MVTx is the only definitive treatment in selected patients with refractory DPMT. We present a series of strategies to decrease the risk associated to this extremely invasive procedure. Preoperative embolization, sequential native organ extraction, standard use of venous-venous bypass and synchronization between donor and recipient teams all contribute to reduce perioperative mortality. MVTx can also be applied in otherwise untreatable extensive intra-abdominal diseases.

## Data Availability Statement

The raw data supporting the conclusions of this article will be made available by the authors, without undue reservation.

## Ethics Statement

The studies involving human participants were reviewed and approved by Institutional Ethics Board of the University Hospitals Leuven (Registration Number: S61313). The patients/participants provided their written informed consent to participate in this study. Written informed consent was obtained from the individual(s) for the publication of any potentially identifiable images or data included in this article.

## Author Contributions

EC, LC, NG, and JP: conception and design. EC and LC: administrative support. IJ, TV, DM, and JP: provision of study materials or patients. EC, LC, NG, GD, and JP: collection and assembly of data. EC, LC, NG, and JP: data analysis and interpretation. All authors: manuscript writing and final approval of manuscript.

## Conflict of Interest

The authors declare that the research was conducted in the absence of any commercial or financial relationships that could be construed as a potential conflict of interest.

## Abbreviations

CIT: Cold Ischemia Time
CT: Celiac Trunk
DPMT: Diffuse PortoMesenteric Thrombosis
GVHD: Graft-Versus-Host Disease
LTx: Liver Transplantation
IVC: Inferior Vena Cava
ITx: Intestinal Transplantation
MELD: Model of End-stage Liver Disease
MVTx: Multivisceral Transplantation
SMA: Superior Mesenteric Artery.
